# Effect of Lignin Content on Cellulolytic Saccharification of Liquid Hot Water Pretreated Sugarcane Bagasse

**DOI:** 10.3390/molecules25030623

**Published:** 2020-01-31

**Authors:** Rafaela I. S. Ladeira Ázar, Sidnei Emilio Bordignon-Junior, Craig Laufer, Jordan Specht, Drew Ferrier, Daehwan Kim

**Affiliations:** 1 Department of Biochemistry and Molecular Biology, BIOAGRO, Federal University of Viçosa, Viçosa, Minas Gerais 36570-000, Brazil; rafaela.ladeira@ufv.br; 2 Laboratory of Biochemistry and Applied Microbiology, São Paulo State University (UNESP), IBILCE, 2265 Cristóvão Colombo, São José do Rio Preto 15054-000, São Paulo, Brazil; bordig@gmail.com; 3 Department of Biology, Hood College, 401 Rosemont Avenue, Frederick, MD 21701, USA; laufer@hood.edu (C.L.); jts14@hood.edu (J.S.); dferrier@hood.edu (D.F.)

**Keywords:** Lignin, sugarcane bagasse, enzymatic hydrolysis, inhibition, bovine serum albumin

## Abstract

Lignin contributes to the rigid structure of the plant cell wall and is partially responsible for the recalcitrance of lignocellulosic materials to enzymatic digestion. Overcoming this recalcitrance is one the most critical issues in a sugar-flat form process. This study addresses the effect of low lignin sugarcane bagasse on enzymatic hydrolysis after liquid hot water pretreatment at 190 °C and 20 min (severity factor: 3.95). The hydrolysis of bagasse from a sugarcane line selected for a relatively low lignin content, gave an 89.7% yield of cellulose conversion to glucose at 40 FPU/g glucan versus a 68.3% yield from a comparably treated bagasse from the high lignin bred line. A lower enzyme loading of 5 FPU/g glucan (equivalent to 3.2 FPU/g total solids) resulted in 31.4% and 21.9% conversion yields, respectively, for low and high lignin samples, suggesting the significance of lignin content in the saccharification process. Further increases in the enzymatic conversion of cellulose to glucose were achieved when the bagasse sample was pre-incubated with a lignin blocking agent, e.g., bovine serum albumin (50 mg BSA/g glucan) at 50 °C for 1 h prior to an actual saccharification. In this work, we have demonstrated that even relatively small differences in lignin content can result in considerably increased sugar production, which supports the dissimilarity of bagasse lignin content and its effects on cellulose digestibility. The increased glucose yields with the addition of BSA helped to decrease the inhibition of non-productive absorption of cellulose enzymes onto lignin and solid residual lignin fractions.

## 1. Introduction

Bagasse is a major residual by-product derived from the fibrous residue of sugarcane stalks in the sugar industry, and it serves as an alternative source for composite, paper, chemicals, second generation energy (ethanol) and other practical agricultural products [1,2,3]. Lignin is an important structural component of plant cell walls and is intricately linked to other structural elements, mainly cellulose and hemicellulose, to provide rigidity to the cell and to prevent against cellular invasion by pathogenic organisms [4,5,6]. The presence of lignin components in the bagasse contributes towards the physical/chemical structure of the plant cells, while unfortunately substantially hampering efficient cellulose conversion into monomeric sugars and the subsequent fermentation process in lignocellulosic biofuel production [4,6,7,8,9,10]. In particular, the physical barrier of lignin prevents enzyme access to cellulose and hemicellulose. In addition, the pretreatment of lignocellulosic biomass to increase access often results in the production of lignin-derived molecules (mainly phenolic acids) that inhibit enzyme activity and/or subsequent downstream processes such as microbial fermentation. The presence of lignin presents another hurdle for efficient enzymatic saccharification of biomass through non-productive binding of the enzymes [8,11,12,13,14].

Pretreatment principally solubilizes hemicellulose and lignin, and reveals inner cellulose molecules that are susceptible to being hydrolyzed by cellulolytic enzymes [15,16]. In addition, substrate particle size, cellulose crystallinity, and cellulose degree of polymerization are decreased during pretreatment, which results increased porosity and surface area that helps digestibility with cellulolytic enzymes [17,18,19,20]. However, pretreatment stimulates the formation of potential inhibitors such as phenols, furan aldehydes, carboxylic acids, and hydrolyzed intermediates that significantly prevent cellulolytic/hemicellulolytic activity for cellulose conversion, as well as microbial viability and fermentative performance [21,22,23,24]. Several studies demonstrated that lignin-derived phenolic molecules are considered as the most powerful cellulolytic inhibitors by causing the non-productive binding of enzymes on the surface of the substrates [21,25,26,27]. For instance, when the lignin-free cellulose (Solka floc) was hydrolyzed in the presence of liquid resulting from pretreated maple (rich in phenols), the cellulose conversion to glucose was decreased by around 50% compared to the yield from a control in the buffer (92% conversion yield) [21]. More recent work reported that 3.5 mg soluble phenols/mg proteins derived from pretreated sugarcane bagasse reduced conversion of Solka floc to glucose by 20%. In addition, further testing with 6.2 mg phenols/mg proteins resulted in a 45% reduction in the conversion yield [28]. In order to alleviate the detrimental effects of lignin and lignin-derived inhibitors on biomass digestion, several different approaches have been pursued. Alriksson and colleagues examined the efficacy of in situ detoxification with reducing agents [29], others have employed activated charcoal [14,16], liquid-liquid extraction [30], lignin-blocking additives (bovine serum albumin or soybean protein) [31,32,33], biological detoxification [13,15] or genetic modification of the lignin [34,35,36,37]. These approaches attack the problem of recalcitrance due to lignin by reducing the concentrations of potential inhibitory molecules, by minimizing the non-productive adsorption of enzymes and/or by reducing concentration of lignin in the biomass to start with. Recent efforts, on the other hand, have found that lignin would also have positive effects on enzymatic degradation of pretreated hydrolysates. Lai et al. [38] elucidated that the removal of extractable lignin from ethanol organosolv-pretreated sweetgum decreased the glucose yields by 6.7%–7.5% compared to the results from control. The extractable lignin, generated during lignin de-polymerization, has a higher S/G ratio and less β-*O*-4, β-β, and β-5 linkages than the residual bulk lignin; the extractable lignin may play a key role in preventing non-productive binding of enzyme onto the surface of residual bulk lignin [38,39]. It is believed the presence of extractable lignin improves the force of electrostatic repulsion between the residual lignin and cellulolytic enzyme that contributes toward enzyme degradation. However, the specific mechanism of extractable lignin was not clearly identified, and the further research into the electrostatic and/or hydrophobic and interactions within enzyme-lignin complexes, still remain to be investigated [38,40].

This study examines the effect of lignin content on enzyme digestion of liquid hot water (LHW) pretreated sugarcane bagasse. Our recent work addressed that breeding of sugarcane crops and their selected clones over a four-year period would change the sugar yield and fiber contents as well as resistance to plant disease [2]. Conventional interbreeding in the closely related sugar cane clones is not expected to produce a unique individual with a large difference in chemical composition; however, two clones with relatively different lignin content were selected for this work [2]. Bagasse from two field-grown sugarcane breeding clones (high *vs*. low lignin composition) was tested for structural changes before/after hydrothermal pretreatment (liquid hot water) and evaluated for subsequent cellulose conversion to glucose at different enzyme loadings. Complex structural change in bagasse samples during pretreatment was analyzed using SEM, and extensive chemical composition was determined at each stage. Moreover, further tests with supplementation of bovine serum albumin, as a non-specific blocking agent, prior to cellulose hydrolysis of pretreated bagasse solids were conducted to examine its efficacy in increasing conversion of cellulose to glucose and enzyme-substrate interaction.

## 2. Results and Discussion

### 2.1. Chemical Composition of Liquid Hot Water Pretreated Sugarcane Bagasse

LHW-pretreatment solubilizes hemicellulose and partially decreases the total lignin content in sugarcane bagasse samples [2,5,41]. The proportion of cellulose (glucan) content in LHW-pretreated samples was increased both in SCBH (from 37.9% to 60.1%) and SCBL (from 36.4% to 63.4%) while the portion of hemicellulose (xylan and arabinan) decreased by 52% and 58% for the high-lignin and low-lignin SCB, respectively. Pretreatment decreased the proportion of lignin by 18% and 21% for high and low lignin SCB, respectively (Table 1). The liquid fraction of the LHW-pretreated bagasse contained inhibitory molecules (Table 2). As anticipated, LHW pretreatment produced a higher concentration of total phenols (656 mg/L) from the high-lignin SCB sample than from the low-lignin SCB (553 mg/L). This difference of 16% of total phenols between the two SCB samples was statistically significant at the level of 95% in a T-test.

It is significant to note that the higher severity factor effectively breaks the glycosidic bonds in hemicellulose, but it causes the lignin recovery through the re-aggregation of lignin and irreversible lignin-degraded fragments that resulted in the increase in toxic soluble inhibitors [2,14]. Michelin et al. [28] elucidated that LHW-pretreatment could solubilize approximately 80% of hemicellulose in sugarcane bagasse at 200 °C for 30 min (severity of 4.42). While there was a small change in lignin content, the treatment resulted in the release of lignin-derived phenolic acids and of compounds with ester groups connecting lignin with hemicellulose. The soluble phenolic compounds derived from the higher severity pretreatment decreased cellulolytic activity to a significantly greater degree than the inhibition resulting from pretreatment with a severity factor of 3.83 [28]. The chemical composition data of SCB here differs from our previous work, where it had higher cellulose (49%–51%), hemicellulose (24%–25%), and lower lignin (23%–25%) components in the initial SCB [8]. This likely reflects the normal variation that will occur under differing growth conditions year to year and field to field. The previous SCB samples were obtained from the crop of 2013. However, the SCB materials utilized in the current work were harvested in 2014, which resulted in different chemical compositions.

### 2.2. Structural Changes in LHW Pretreated Solids

**Figure 1:**
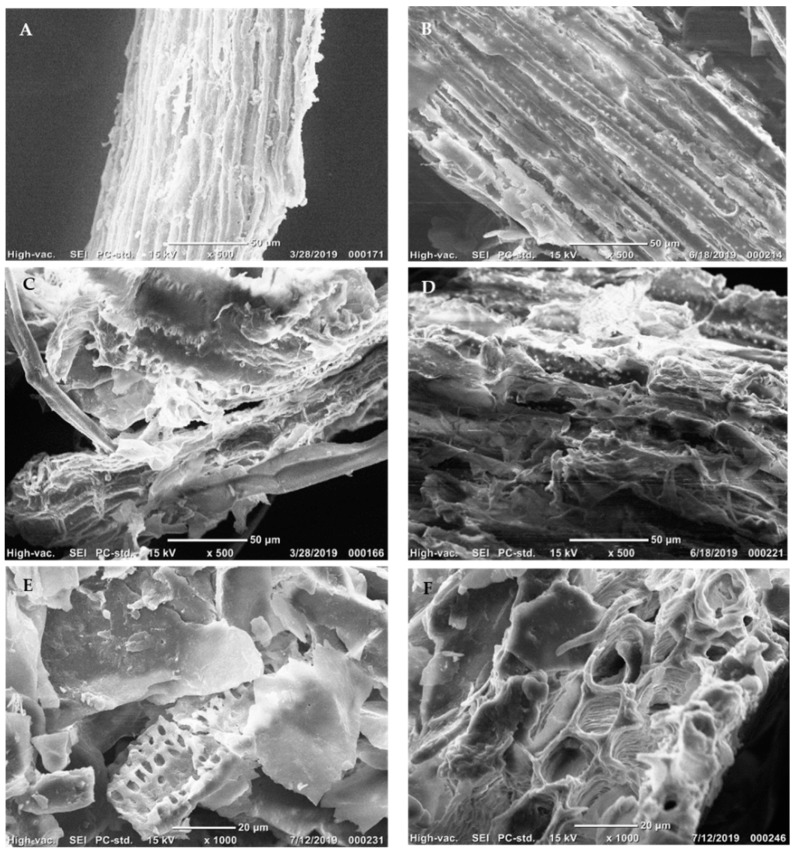
Scanning electron micrograph (SEM) of raw sugarcane bagasse, liquid hot water pretreated solids, and enzymatically hydrolyzed solids. (**A**) Untreated SCBH and (**B**) SCBL; (**C**) liquid hot water pretreated SCBH and (**D**) SCBL at 190 °C for 20 min (Severity factor: 3.95); (**E**) LHW-pretreated, washed and hydrolyzed SCBH and (**F**) SCBL using 10 FPU enzyme/g glucan. The morphological changes in each sample were captured at 500× or 1000× magnification. The scale bar and magnification levels are presented in each picture.

To evaluate the changes in the pretreated solids, the morphological appearances were captured by scanning electron micrograph (SEM). The surfaces of SCBH and SCBL were not damaged by the crushing and grinding step, showing an intact structure with a rigid and fibrillary morphology in untreated samples (Figure 1A,B). In contrast, the samples of LHW-pretreated bagasse were broken down and fragmented (Figure 1C–F). When treated in hot water at 190 °C for 20 min, the highly compact and smooth surface of untreated bagasse was disrupted with a large number of irregular lamellar fibers and fragments (Figure 1C,D).

In general, it is found that plant cell walls coated with lignin and treated at over 160 °C, causes the lignin to melt and complex internal components (mainly cellulose and hemicellulose) be disrupted and/or solubilized with some degraded molecules while causing greater exposure of cellulose to the outer region of the cell walls, offering more binding sites for enzymatic reactions [16,31,32]. These morphological changes with the removal of hemicellulose and lignin contribute to enhancing the accessibility of enzymes onto the surfaces of the cellulose. The most remarkable alteration in hydrolyzed solids was the appearance of coarse surfaces and the formation of small pores in the internal structure (Figure 1E,F). It is possible that the exposed cellulose was degraded with enzymes into smaller molecules, e.g., sugars, and the separated fibrillary matrix structure was presented with the inter-fibrillar parenchyma and vascular bundles [12].

The LHW pretreatment step not only affects the morphological change in biomass, but also influences the generation of inhibitors, which may deactivate/inhibit action of enzymes for cellulose conversion. However, in this study, many of the inhibitory molecules produced during LHW pretreatment were removed from the SCB by extensive washing and separation of the solids from the liquids. Nevertheless, the remaining lignin and/or lignin derived byproducts still contribute to biomass recalcitrance due to the non-productive adsorption of cellulases to the exposed lignin. To explore that possibility, we examined the saccharification of the SCBH and SCBL samples in the presence and absence of an additional pretreatment of added BSA, which is proposed to block the non-specific protein binding sites of the lignin and therefore should enhance saccharification.

### 2.3. Cellulose Hydrolysis in LHW-Pretreated Sugarcane Bagasse

**Figure 2:**
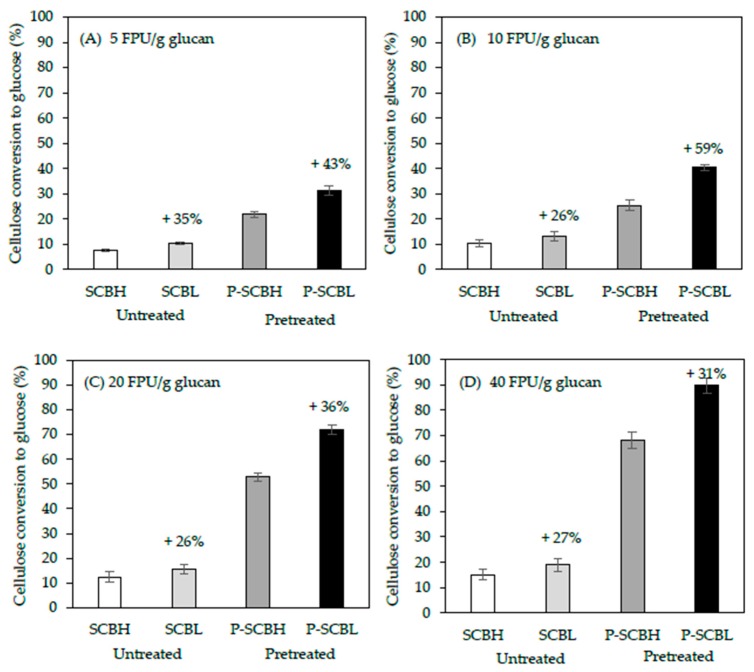
Effect of enzyme loadings (5–40 FPU/g glucan) on enzymatic hydrolysis of untreated raw materials and liquid hot water pretreated SCB at 190 °C for 20 min (Severity factor: 3.95): (**A**) 5 FPU/g glucan, (**B**) 10 FPU/g glucan, (**C**) 20 FPU/g glucan, and (**D**) 40 FPU/g glucan. The increase of enzyme loading for two pretreated bagasse samples was applied for glucan conversion to glucose. All runs were duplicated and data were analyzed with 95% significant difference. SCBH: sugarcane bagasse high lignin content; SCBL: sugarcane bagasse low lignin content; P-SCBH: pretreated-sugarcane bagasse high lignin content; P-SCBL: pretreated-sugarcane bagasse low lignin content.

In order to assess the effects of lignin content in bagasse samples on the enzymatic conversion of cellulose to glucose, responses to various intensities of cellulase were evaluated and compared. Enzymatic saccharification was carried out using enzyme dosages of 5, 10, 20, and 40 FPU/g glucan of Cellic Ctec2 at 50 °C for 72 h in a shaking incubator with 250 rpm. The percent conversion of cellulose to glucose was a function of the enzyme concentration (Figure 2A–D). The LHW pretreatment significantly improved the total yield of saccharification in both SCBH and SCBL samples as we expected (Figure 2A–D). The highest glucan conversion yield (89.7%) was observed in the pretreated SCBL at 40 FPU enzyme loading (Figure 2D). At all four enzyme concentrations tested, for both untreated and pretreated SCB, the low-lignin samples produced greater conversion to glucose than the high-lignin samples. At enzyme treatments of 40 FPU/g glucan, for the LHW samples, there was a 31% increase in the conversion of cellulose to glucose (68.3% to 89.7%) in the SCBL sample compared to the SCBH sample. Similarly, at the lowest enzyme concentration tested, 5 FPU/g glucan, there was a 43% increase in conversion in the SCBL compared to SCBH (21.9% to 31.4%). The magnitude of the difference between low and high-lignin samples is highlighted by the finding that the percent cellulose conversion of pretreated SCBL at 20 FPU/g glucan is greater than the cellulose conversion of SCBH treated with 40 FPU/g glucan cellulase (Figure 2C,D).

These results indicate that the SCBL was more susceptible to enzymatic degradation with less recalcitrance compared with the SCBH, likely due to the higher lignin composition in SCBH. These results are in agreement with the earlier study, which highlighted that the downregulating of lignin biosynthesis in alfalfa not only increased the total carbohydrate levels in biomass, but its hydrolysis after pretreatment largely improved the saccharification efficiencies (67%–79% in lignin reduced lines *vs*. 43% in the control) [42]. More recent work with the genetically manipulated switchgrass in the caffeic acid O-methyltrasferase (COMT) gene also points to the utility of lowering lignin content to enhance saccharification yields. For example, there was a remarkable increase in conversion efficiency of 17%–22% and improved sugar release by up to 34% after dilute acid and liquid hot water pretreatment, respectively [36,43]. It is worthwhile to note that decreasing lignin content and/or changing lignin composition (S/G ratio) has positive correlations to reduce lignin recalcitrance and to enhance the enzymatic digestion of lignocellulosic biomass and fermentable sugar yields [35,37,42,44].

### 2.4. Effect of Bovine Serum Albumin (BSA) in Enzymatic Hydrolysis

**Figure 3:**
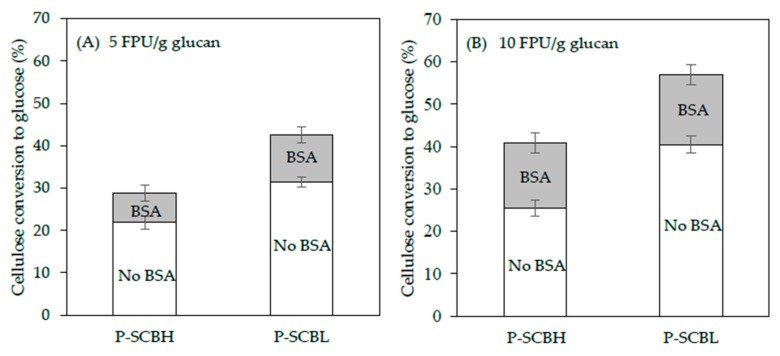
Comparison of glucan conversion of liquid hot water pretreated SCBH and SCBL solids at 1% (*w*/*v*) glucan concentration in 50 mM citrate buffer solution (pH 4.8) with either 5 FPU or 10 FPU/g glucan Cellic Ctec2. The hydrolysis was carried out at 50 °C for 72 h with agitation of 250 rpm.

The generation of inhibitory molecules from lignocellulosic feedstock is dominantly related to the raw material type, solid concentration, pretreatment method and its severity. In general, more sever pretreatments result in greater access for saccharification enzymes but at the cost of greater production of undesirable, inhibitory by-products [27,32,45]. The presence of inhibitors, both soluble, and in the solid fractions result in non-productive enzyme binding onto the surface of cellulose, and inhibition/deactivation of the cellulolytic enzyme reaction [21,25,26]. To alleviate the non-productive binding of the catalytic enzyme, BSA (non-catalytic protein, 50 mg BSA/g dry solids) was added to the LHW pretreated SCB and pre-incubated at 50 °C for 1 h with shaking at 250 rpm prior to actual enzymatic saccharification with Cellic Ctec 2. The hydrolysis was performed at 5 and 10 FPU enzyme/g glucan, respectively, and the glucose yields were compared with the results from samples without BSA (Figure 3). The difference in saccharification efficacy between high/low lignin content and with/without BSA in Figure 3 was statistically different with a confidence level of 95% by the T-test (n = 2). The supplementation of BSA enhanced the cellulose conversion yields in both SCBH and SCBL at both cellulase concentrations tested. For the treatments with cellulase at 10 FPU/g glucan, the addition of BSA increased the conversion of cellulose to glucose by 60% for SCBH (from 26% to 41%) and by 40% for SCBL (from 41% to 57%). On the other hand, for the 5 FPU/g glucan cellulase reactions BSA treatment increased the yield of glucose by similar amounts, 32% for SCBH and 35% for SCBL (Figure 3A,B). These results are consistent with our earlier findings that lignin-dependent, non-soluble factors negatively influence the catalytic performance of cellulolytic enzymes and decreased conversion yields [2,8,14]. What seems remarkable here is that these effects are clearly evident in the differences between SCBH and SCBL even though the difference in lignin contents between the pretreated SCBH and SCBL is quite small (22.3% *vs*. 20.6%, Table 1). It should also be noted that our experiments used LHW-treated SCB that was washed prior to the saccharification reactions. Unwashed solids samples contain a higher level of soluble inhibitors such as phenolic compounds and acidic molecules. Washing of hydrothermally pretreated residual samples improve the lignocellulose conversion to monomeric sugars [1,4,6,15,16]. For instance, our previous study presented that remarkable improvement of glucose conversion was observed when the residue sample was washed using hot water prior to enzyme hydrolysis (40%–43% unwashed samples *vs*. 63%–81% washed samples) [8]. As shown in Table 2, the pretreatment of SCBH produced more potential soluble enzyme inhibitors, particularly total phenols, than the pretreated SCBL.

These inhibitors would largely be removed by our washing and, therefore, not be a factor in our results. However, for pretreated biomass that was not washed prior to the saccharification reaction, these inhibitory molecules would be present. Thus, the advantages of using SCB from low-lignin strains would potentially be even greater than shown here.

We propose that the use of BSA contributed to minimizing enzyme adsorption on lignin solids allowing the cellulolytic enzymes to spend more time with the cellulose substrate resulting in increased conversion yields. Interestingly, for the 10 FPU/g glucan cellulase treatment, the addition of BSA enhanced the glucose yield to a greater extent, by 60%, over the non-BSA treated sample for the high-lignin substrate compared to the low-lignin substrate (40%). This might be expected as the issue of non-productive adsorption to lignin would be more severe in the higher lignin substrate. However, this was not evident in the treatment with 5 FPU/g glucan enzyme, suggesting that, at different cellulase concentrations, the relative importance of the different mechanisms leading to enzyme inhibition vary.

The results presented in this paper confirm that a higher lignin content directly suppresses cellulolytic enzyme protein to substrates while additional supplement of non-cellulolytic protein result in the reduction of non-productive binding which in turn enhance cellulose activity or cellulose conversion. It should be noted that while the current work has focused on evaluating the effect of lignin content in different bagasse clones, their inhibitory influence on the cellulose conversion, and the alleviation of non-specific binding of the enzymes with BSA, the saccharification efficacy found here could be further improved with other factors such as the pretreatment method, severity factor, cellulolytic enzyme preparation, types of lignin-binding chemicals and their performing conditions (concentration, pre-incubation time, and temperature). For instance, Florencio et al. [12] found that the addition of 12% (*w*/*v*) soybean protein (less expensive lignin-blocking agent) remarkably increased glucose release (76% higher) from the LHW-pretreated SCB at a high solid concentration of 15% (*w*/*v*) using 5 FPU/g dry solids. Therefore, we expect that there is still room for improvement in terms of both yield and minimizing the expense of converting reduced lignin SCB biomass into value-added products.

## 3. Materials and Methods

### 3.1. Materials

Raw sugarcane bagasse (SCB) from high lignin (SCBH) and low lignin (SCBL) clones originated from the breeding program carried out by the Center Research and Breeding Sugarcane, and they were kindly provided by the Federal University of Viçosa, Brazil. The SCB samples used for this study were field-grown in the experimental field from Oratórios (20°25′5′’ latitude south, 42°47′28′’ longitude east) and cultivated in 2014. They were washed with distilled water, oven dried overnight at 50 °C and ground in a Wiley hammer mill (Thomas Scientific, Werdesboro, NJ, USA) with a 20-mesh screen (0.84 mm). The sieved sample was further dried at 50 °C until reaching a constant weight and then pretreated via liquid hot water. The moisture content of samples was 4.0% (Halogen moisture analyzer, Mettler Toledo HB43, Columbus, OH, USA). The chemical composition of the SCB before and after pretreatment is presented in Table 1 and Table 2. Cellic Ctec 2, cellulase/hemicellulose preparation (Novozyme, North America INC., Franklinton, NC, USA), was used for the enzymatic saccharification of the sugarcane bagasse sample. Enzyme activities of a filter paper unit (FPU, 90.1 IU/mL), endo-glucanase (195.8 U/mL), β-glucosidase (206.7 U/mL), and protein (150 mg/mL) concentration were determined in our laboratory [14,46].

### 3.2. Liquid Hot Water Pretreatment

Sugarcane bagasse (1.0 g dried biomass in 10 mL distilled water, 10% *w*/*v*) in stainless steel reaction tubes was pretreated at 190 °C for 20 min (severity factor: 3.95) as previously described [1,5,16,47]. The resulting pretreated slurry was vacuum filtered through a #1 Whatman filter paper (Whatman International Ltd., Springfield, England, Cat. No. 1001125) to separate solids and the liquid fraction. The separated solids were washed three times with 30 mL of distilled water at room temperature, oven-dried at 50 °C overnight, and stored at room temperature for further use. The severity factor of 3.95 was determined with pretreatment temperature (T) and time (t) by the equation log *R*_0_ = log t × exp ((T−100)/ω), where ω = 14.75 (the conventional activation energy, kJ/mol), which was applied to liquid hot water pretreatment and provided a linear correlation for sugarcane bagasse [5,48].

### 3.3. Compositional Analysis

The composition of raw and liquid hot water (LHW)-pretreated bagasse solids was determined following the National Renewable Energy Laboratory (NREL) LAP standard analytical procedures [49]. All compositional analyses were performed in triplicate. The SCB samples were statistically analyzed by a *t*-Test with a confidence difference of 95%, for enzymatic saccharification of raw/pretreated and lignin content in SCB samples (Minitab 16 software, Minitab Inc., State College, PA, USA). The total phenolic compounds in the hydrolysate samples were measured by spectrophotometer at 765 nm with a Folin-Ciocalteu colorimetry assay [8].

### 3.4. Enzymatic Hydrolysis of Sugarcane Bagasse

A total of 10 mL of 50 mM citrate buffer (pH 4.8) and Cellic Ctec2 were mixed with pretreated SBC, 1% (*w*/*v*) glucan, in a 50 mL flask, and incubated in a shaker at 50 °C and 250 rpm for 72 h. The enzyme was added at 5, 10, 20, and 40 FPU/g glucan, respectively. After hydrolysis, each sample was centrifuged for 5 min at 13,000 rpm (7500× *g*), and the liquid fraction was filter-sterilized with a nylon syringe filter (0.45 µm filter, Acrodisc, Cortland, NY, USA) and kept at 4 °C for later analysis of remaining enzyme activity, and the protein and glucose concentrations. All experiments were performed in duplicate.

### 3.5. Scanning Electron Microscopy (SEM)

The structural change in LHW-pretreated bagasse solids was observed using a JCM-6000 benchtop SEM (JICM 6000-OG-2, model number: MP 1220004340434, JCM, Peabody, MA, USA). A small amount of oven-dried biomass was attached to the aluminum stub with double-sided adhesive carbon tape and sputter coated with AuPd using a Cressington 108 sputter coater (serial number: C6119, Ted Pella Inc., Redding, CA, USA). The prepared samples were imaged at 15 kV and a magnification range of 500× to 1000×.

### 3.6. Analytical Assays

Cellulolytic activities in Cellic Ctec2 were determined with 1% carboxylmethyl cellulose sodium salt (CMC, Sigma-Aldrich, St. Louis, MO, USA) and 10 mM *p*-nitrophenyl-β-d-glucopyranoside (pNG, Sigma-Aldrich, St. Louis, MO, USA) as substrates for endo-glucanase and β-glucosidase, respectively [46]. One unit of enzyme activity refers to the enzyme amount that releases 1 micromole of the specified substrate per min under described protocol conditions [46]. The glucose content of hydrolyzed samples was quantified using a d-glucose GOPOD-format assay kit (Megazyme, Wicklow, Ireland). The soluble inhibitors of furfural, hydroxymethyfurfural (HMF), and acetic acid content were quantified by HPLC as described in our previous work [14,15]. The HPLC system employed an Aminex HPX-87H ion exchange column (300 × 7.8 mm, Bio-Rad Laboratories Inc., Hercules, CA, USA) and was equipped with a refractive index detector.

## 4. Conclusions

The disruption of a complex crystalline structure of raw bagasse samples, exposed internal chains of cellulose and hemicellulose, and morphological changes after enzymatic saccharification were confirmed by SEM (Figure 1). The changes in physical structure, lignin content and composition considerably impact the cellulose conversion of the liquid hot water pretreated sugarcane bagasse. In particular, the reduction of intermolecular and intramolecular linkages/interactions in the crystalline portion of cellulose contributed to the susceptibility and accessible surface area of cellulose to cellulolytic enzymes that improved the conversion yield at low enzyme dosages [1,2,4]. Even a small difference in lignin composition in SCBH and SCBL (22.3% *vs*. 20.6%) resulted in significant differences in cellulose to glucose conversions. Hydrolysis tests with SCBL gave higher yields up to 89.5% compared to the SCBH of 68.3%. Digestions with lower enzyme loadings confirmed the efficacy of adding BSA to diminish the non-specific binding of cellulolytic enzymes on lignin solids to improve the saccharification efficiency.

## Figures and Tables

**Table 1:** Compositional analysis of sugarcane bagasse samples before and after pretreatment by liquid hot water pretreatment. For the LHW pretreatment, 10% (*w*/*v*) sugarcane bagasse materials with high (SCBH) and low (SCBL) lignin content were pretreated in liquid hot water at 190 °C for 20 min (Severity factor: 4.56). Compositional analysis was done in triplicate.

Composition (%)	Bagasse High Lignin		Bagasse Low Lignin	
	Raw SCB, Untreated	LHW-Pretreated	Raw SCB, Untreated	LHW-Pretreated
Glucan	37.94 ± 0.2	60.14 ± 0.42	36.44 ± 0.03	63.44 ± 0.03
Xylan	18.39 ± 0.15	8.38 ± 0.19	17.91 ± 0.06	6.88 ± 0.07
Arabinan	3.18 ± 0.02	2.05 ± 0.01	3.21 ± 0.05	1.98 ± 0.02
Lignin	27.2 ± 0.39	22.28 ± 0.24	26.14 ± 0.04	20.55 ± 0.34
Acetyl	9.84 ± 0.15	4.65 ± 0.07	9.97 ± 0.19	4.53 ± 0.29
Ash	2.93 ± 0.2	3.45 ± 0.12	4.29 ± 0.08	2.8 ± 0.52
Total	99.48 ± 0.19	100.95 ± 0.18	97.96 ± 0.08	100.18 ± 0.21

**Table 2:** Soluble inhibitors in liquid fraction after vacuum filtration.

Soluble Inhibitors	Composition of Vacuum Filtrate	
^a^ Furfural (g/L)	2.8 ± 0.48	2.4 ± 0.74
^a^ Hydroxymethylfurfural (HMF) (g/L)	0.12 ± 0.02	0.11 ± 0.03
^a^ Acetic acid (g/L)	1.82 ± 0.31	1.74 ± 0.39
^b^ Total phenols (mg/L)	655.76 ± 3.39	552.73 ± 6.41

^a^ High performance liquid chromatography analysis. ^b^ Folin-Ciocalteu colorimetry assay.
